# Visualizing Non Infectious and Infectious *Anopheles gambiae* Blood Feedings in Naive and Saliva-Immunized Mice

**DOI:** 10.1371/journal.pone.0050464

**Published:** 2012-12-13

**Authors:** Valerie Choumet, Tarik Attout, Loïc Chartier, Huot Khun, Jean Sautereau, Annie Robbe-Vincent, Paul Brey, Michel Huerre, Odile Bain

**Affiliations:** 1 Unité de Biochimie et de Biologie Moléculaire des Insectes, Institut Pasteur, Paris, France; 2 Unité de Parasitologie, UMR 7205 CNRS, Muséum National d'Histoire Naturelle, Paris, France; 3 Unité de Recherche et d'Expertise Epidémiologie des Maladies Emergentes, Institut Pasteur, Paris, France; 4 Unité de Recherche et d'Expertise Histotechnologie et Pathologie, Institut Pasteur, Paris, France; Metabiota, United States of America

## Abstract

**Background:**

*Anopheles gambiae* is a major vector of malaria and lymphatic filariasis. The arthropod-host interactions occurring at the skin interface are complex and dynamic. We used a global approach to describe the interaction between the mosquito (infected or uninfected) and the skin of mammals during blood feeding.

**Methods:**

Intravital video microscopy was used to characterize several features during blood feeding. The deposition and movement of *Plasmodium berghei* sporozoites in the dermis were also observed. We also used histological techniques to analyze the impact of infected and uninfected feedings on the skin cell response in naive mice.

**Results:**

The mouthparts were highly mobile within the skin during the probing phase. Probing time increased with mosquito age, with possible effects on pathogen transmission. Repletion was achieved by capillary feeding. The presence of sporozoites in the salivary glands modified the behavior of the mosquitoes, with infected females tending to probe more than uninfected females (86% versus 44%). A white area around the tip of the proboscis was observed when the mosquitoes fed on blood from the vessels of mice immunized with saliva. Mosquito feedings elicited an acute inflammatory response in naive mice that peaked three hours after the bite. Polynuclear and mast cells were associated with saliva deposits. We describe the first visualization of saliva in the skin by immunohistochemistry (IHC) with antibodies directed against saliva. Both saliva deposits and sporozoites were detected in the skin for up to 18 h after the bite.

**Conclusion:**

This study, in which we visualized the probing and engorgement phases of *Anopheles gambiae* blood meals, provides precise information about the behavior of the insect as a function of its infection status and the presence or absence of anti-saliva antibodies. It also provides insight into the possible consequences of the inflammatory reaction for blood feeding and pathogen transmission.

## Introduction

*Anopheles gambiae* sensu lato (s.l.) consists of seven mosquito species, including *An. gambiae* sensu stricto (s.s), *An. arabiensis* and *An. melas*, three of the major vectors of lymphatic filariasis (LF) and malaria, which are caused by *Wuchereria bancrofti* and *Plasmodium falciparum*, respectively, in West Africa. The role of *Anopheles* mosquitoes as vectors of both human malaria and LF has long been established, but the prevalence of concomitant infections in a single mosquito vector has been reported to be rare in nature [1], [2]. *Wuchereria*-infected *An. gambiae s.l.* mosquitoes have been shown to have significantly higher rates of infection with *Plasmodium falciparum* sporozoites than uninfected mosquitoes.

The fight against vector-borne parasitic diseases is based on mosquito control and the use of parasiticidal medicines. However, resistance to insecticides and anti-parasitic drugs is on the rise, increasing the already intolerable burden of these diseases in the countries in which these diseases are endemic. Studies aiming to improve our understanding of vector/parasite/host interactions would clearly constitute a major step forward in efforts to disrupt parasite transmission. One of the key steps in disease transmission is vector interaction with the skin. Studies of the steps involved in this contact would facilitate identification of the mosquito and host factors important for effective parasite transmission and, thus, of innovative targets for the control of these tropical diseases.

Parasites are transmitted to the host or the vector during a blood meal taken by an adult female mosquito to provide the necessary resources for egg development. The saliva of the mosquito plays a key role in overcoming the challenges posed by the host: pain and itch responses, immune defenses and hemostasis [3], [4]. Not all pathogens, notably the filariae, are transmitted directly from the salivary glands of infected arthropods to vertebrate hosts, but the saliva of the vector is nonetheless thought to be an essential factor in disease transmission, either increasing the infectiousness of the parasites or attenuating the host immune response [5], [6].

The host selection behavior of mosquitoes has been studied both in the field and in the laboratory [7], [8], [9]. However, it is more difficult to investigate what happens in the skin during the bite itself and the mechanism by which these vectors suck blood from the host. Moreover, several observations have suggested that the pathogen may be able to modify the feeding behavior of the vectors, lengthening the duration of the probing phase, as shown for malaria transmission [10], [11], or increasing the mean number of bites, as demonstrated for *Trypanosoma rangeli* infection of *Rhodnius prolixus* [12] or the number hosts bitten by the *Plasmodium*-infected mosquito to achieve complete repletion [10], [13]. The changes in host behavior induced by parasites are of epidemiological importance if they affect the rate of parasite transmission.

Previous observations of mosquito blood feedings have focused on *Aedes aegypti* mosquitoes feeding on the leg of a frog or the ear of a mouse [14], [15]. The path followed by the mosquito's mouthparts under the skin was explained with photographs and drawings. In this study, we studied the behavior of *Anopheles gambiae* and its consequences for mouse skin physiology and parasite transmission. We used *Plasmodium* as our model organism for studies of pathogen transmission. Malaria affects 40% of the world's population, in tropical and subtropical regions. A mouse model of infection with this parasite is available and was used in this study [16]. We used intravital videomicroscopy to analyze the feeding behavior of *Anopheles gambiae*. We observed the mosquito feeding through the skin of the back of an anesthetized mouse, as described by Petit [17]. The mice were either naive, or had been passively or actively immunized with *Anopheles gambiae* saliva. The reaction of the skin to *Anopheles gambiae* blood feedings was followed over time by histological observation. Immunohistochemistry was used to localize the release of saliva and sporozoites, and to follow the course of saliva and sporozoite detection in the skin.

## Methods

### Ethics statement

All studies on animals followed the guidelines on the ethical use of animals from the European Communities Council Directive of November 24, 1986 (86/609/EEC). All animal experiments were approved and conducted in accordance with the Institut Pasteur Biosafety Committee. Animals were housed in the Institut Pasteur animal facilities accredited by the French Ministry of Agriculture to perform experiments on live mice, in appliance of the French and European regulations on care and protection of the Laboratory Animals (accreditation number B 75 15-01 and B 75 15-07). The study protocols were approved by the Comité d'Ethique pour l'Expérimentation Animale (CEEA) - Ile de France - Paris - Comité 1.

### Mosquitoes and infection

The *Anopheles gambiae* mosquitoes used belonged to a strain from Yaoundé (Cameroon) reared in our insectarium. This strain is maintained in the laboratory for more than 30 generations. Mosquitoes were infected by feeding on mice injected with the NK65 strain of the *Plasmodium berghei* parasite expressing GFP protein at the oocyst and sporozoite stages [18].

### Exposure of mice to mosquitoes

Mice (Swiss mice, 4 to 8 weeks old, Janvier) were anesthetized by an i.p. injection of ketamine (600 mg/kg) and xylazine (20 mg/kg) and placed on top of mosquito cages to allow the mosquitoes to bite them through the netting. Mosquito bites (50 mosquitoes per cage) were focused on a 2 cm^2^ area of skin on the back delimited with paper tape. Mice were either naive before exposure to mosquito bites (for histology and real-time observation) or were subjected to four feeding sessions at one-week intervals (active immunization) and/or injected with 200 or 400 µg of IgG antibody against saliva prior to contact with mosquitoes (for real-time observation). All experiments were approved by the institutional review board of Institut Pasteur and were carried out in accordance with the international guidelines and regulations.

### Kinetics of fluid extravasation after the mosquitoes' blood feeding

Mice were exposed to mosquitoes as described above and were sacrificed at various time points (30 min, 1 h, 3 h, 5 h, 8 h, 16 h, 24 h, 48 h). Thirty minutes before the anesthetized animals were killed, Evans blue dye (1% in PBS; 50 µl/mouse) was injected into the retro-orbital vein. The skin from the back was removed for examination and photography of the inner side. Bites were easily detectable as hemorrhagic spots of 0.5 to 1 mm in diameter in the hypodermis.

### Immunization of mice by mosquito bites and analysis of the immune response by ELISA

Rabbits were exposed to mosquito bites two times a week for several weeks. Blood was drawn and the reactivity of rabbit sera against *Anopheles gambiae*'s saliva was tested by ELISA at different times post-feeding. When a plateau was reached, rabbits were bled and immunoglobulins were isolated using the Melon kit (Thermo LifeScience, Rockford, Il).

Mice were exposed to mosquitoes once per week, for four weeks. Blood samples were taken at the end of these sessions and sera were isolated and used for ELISA-based tests of the reaction to *Anopheles gambiae* salivary components. For this purpose, salivary glands from *Anopheles gambiae*'s females were sonicated for 20 min at 4°C as described in [19]. Microtitration plates were coated with salivary gland extract (5 µg/ml in PBS), by incubation overnight at 4°C. The plates were saturated by incubation with 100 µl of 3% BSA in PBS for 1 h at 37°C, and then incubated for 1 h at 37°C with serial dilutions of mouse serum in 3% BSA in PBS supplemented with 0.1% Tween 20. The plates were then washed and incubated with peroxidase-conjugated anti-mouse IgG for 1 h at 37°C. The plates were washed again and incubated with orthophenylene diamine, a peroxidase substrate, for the detection of antibody binding. Mice with sera reacting against saliva (optical densities of 0.8 to 1) were used at least one week after the last session of bites.

### Real-time observation of blood meals in mice

Mice were anesthetized (i.p. injection of 150 µl of a solution containing 150 µl Imalgen (ketamine), 20 µl Rompun (xylazine) and 830 µl PBS) and sacrificed at the end of the experiment, in accordance with the international guidelines and regulations. The anesthetized mice were shaved and a section of skin from the back was cut such that three of its four sides were free, and detached from the first muscle layer. Mice were placed on one side on the platform and the detached piece of skin was placed on a microscope slide and viewed under the objective of a Nikon Eclipse TE200 reverse microscope. The mosquito under observation was confined to a circular piece of glass tubing 10 mm in diameter and 10 mm high. A circular window covered by mosquito netting on its upper surface provided the mosquito with access to the skin of the mouse. A digital color video camera (Sony Exwave HAD) was used to record all stages of the blood meal. We calculated the time until the insect began to probe, the duration of probing and the duration of the blood meal from real-time observations. We also defined the type of blood-feeding: capillary or pool feeding, vessel diameter (defined as a function of the diameter of the proboscis). The temperature in the room remains similar for all experiments and ranged from 18 to 22°C. In experiment implying infected and uninfected mosquitoes, the two groups were always compared the same day and different mice were used for the same group of mosquitoes to overcome the intra-animal variation. When immunized mice were tested, control mice were always used to compare the results with the same series of mosquitoes and the same environmental condition.

### Histology and immunohistochemistry

Histological analysis was performed on each mouse. We removed a portion of skin from sacrificed mice 2, 6, and 16 hours after the mosquito bite, and fixed it in freshly prepared 4% formaldehyde solution (pH 7.4). Fixed tissues were embedded in low-melting point fusion paraffin, cut into 3 µm–thick sections and serial slices of the same portion of skin were stained with hematoxylin and eosin (H&E), Giemsa stain for light microscopy, and with anti-saliva and anti- circumsprorozoite protein antibodies.

For immunohistochemistry, sections were incubated with the primary antibodies diluted in permeabilization buffer (1 mg/ml bovine serum albumin and 0.05% saponin in PBS) for 1 hour. Rabbit polyclonal antibodies directed against *Anopheles gambiae* saliva and a monoclonal antibody directed against *Plasmodium berghei* circumsprorozoite protein (CSP, Abgene) were used as primary antibodies. Tissues were washed (3×5 minutes) in the permeabilization buffer, and proteins were detected by incubation of the tissues for 1 hour with peroxidase-conjugated goat anti-mouse or anti-rabbit IgG (H+L) antibodies from Cappel Laboratories.

### Statistical analysis

We compared the various groups of mosquitoes defined on the basis of infection status, age, infection status of the mice that were bitten or the active or passive immunization of the mouse with *Anopheles gambiae* saliva or salivary gland extract. Data are expressed as medians and interquartile ranges or mean ± standard deviation for continuous variables and percentages for discrete variables. Univariate analyses were carried out, with Fisher's exact test used for discrete variables and the Mann-Whitney test used for continuous variables. All baseline variables associated with outcome in univariate analysis (p<0.25) were included in a backward stepwise logistic regression model. The likelihood ratio method was used for significance testing. A p value of <0.05 was considered to denote statistical significance. Data were analyzed with STATA software version 12.0 (Stata Corporation, College Station, Texas).

## Results

### Videomicroscopy observations of probing and blood-feeding on mice by *Anopheles gambiae* mosquitoes

In total, 300 observations were made with the equipment shown in Figure S1. In these observations, complete or partial feeding was observed on 200 occasions (66%). Thirty three percent of the mosquitoes did not probe at all. Figure S2A shows a mosquito inside the glass tube during engorgement and Figure S2B shows the same mosquito after blood feeding. A small drop of blood has been discharged from the anus of the mosquito onto the mouse skin.

The process of blood-feeding can be divided into two steps. The first is the probing phase, during which the arthropod seeks a blood vessel. It is during this period that saliva is released below the skin, to counteract physiological responses to the arthropod, such as hemostasis and inflammation. Once a blood vessel has been found, the engorgement step begins. This phase continues until complete repletion of the arthropod is achieved. We will review the behavior of *Anopheles gambiae* during these two phases through still photographs and intravital movies.

#### Probing phase and blood feeding

**Figure 1:**
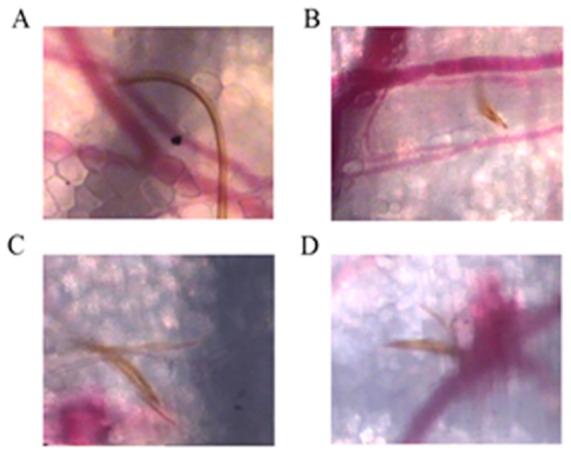
Intravital micrographs illustrating different steps in the blood meal. A and B: flexibility of the labrum; C: mouthparts within the skin, showing the labrum and maxillae in particular; D: damage due to the passage of the proboscis through a blood vessel during probing, triggering blood extravasation.

The mosquito searched for a suitable site for penetration of the skin by the proboscis. In a number of cases, probing began immediately, but in some observations, the proboscis was moved about over the mouse skin for some time before the fascicle penetrated the skin. In some cases, the mosquitoes refused to probe and remained on the side of the glass tube, completely ignoring the presence of the mouse (25% to 40% depending on the status of the mosquito or of the mouse). By following the movement of the labella, which can be recognized as a dark shadow moving around on the surface of the skin, we were able to localize the point of entry of the fascicle and to observe the probing that subsequently occurred. For most bites, multiple probing (3 to 5) was observed (80%). Figures 1A and B illustrate the considerable capacity of the tip of the fascicle to bend. The labrum and the other stylets were observed to progress into the dermis of the skin (Movies S1 and S2, Figure 1C). In Movie S1, the bevelled apex of the labrum was readily observable, whereas one of the maxillae had clearly separated from the other one. The blade of the maxillae can be seen, with its lateral teeth. One of the maxillae moved rapidly on one side of the labrum. In Movie S2, the mouthparts have clearly separated within the dermis, with one of the maxillae and the two mandibles visible in the top right corner and the labrum and one of the maxillae visible in the bottom left corner.

Some mosquitoes probed areas completely devoid of blood vessels, continuing for several seconds before withdrawing the stylets to find another area to probe (10%). We tried to determine whether this behavior was due to the experiment itself. Indeed, in our experimental conditions, some of the mouse skin was cut off from the body, with only one side of the portion examined remaining attached to the body. This might have decreased the temperature of the skin, modifying blood sensing. We therefore also used the ears of the mice as a control, and similar results were obtained (data not shown).

Movie S3 shows a probing that triggered local tissue damage, as shown in Figure 1D. The fascicle passed through a blood vessel as soon as it entered the dermis and its withdrawal resulted in an extravasation of blood. On subsequent images, some red blood cells were visualized in the food canal, indicating that the insect had detected the pool of blood and sucked some of it up, but it nevertheless continued probing.

#### Salivation

**Figure 2:**
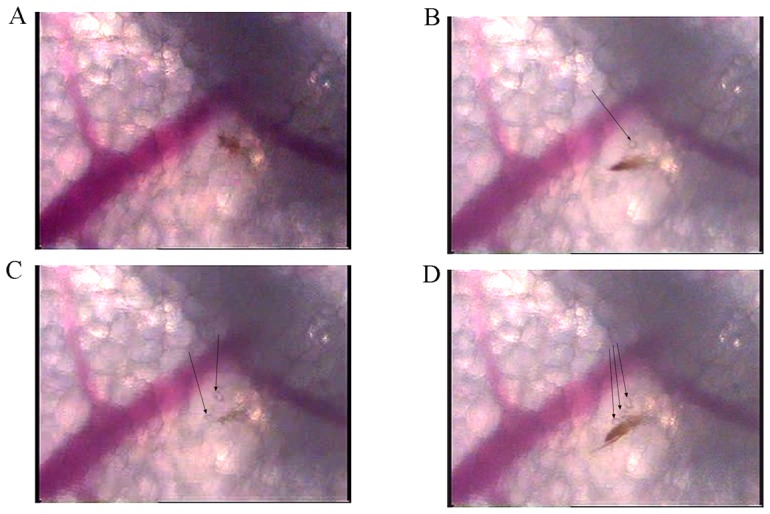
Intravital micrographs illustrating probing with salivation. Series of bubbles were observed at each movement of the proboscis (A to D), as shown by arrows.

A series of bubbles surrounded the tip of the labrum during each of its movements in an avascular area (Figures 2A to D, Movie S4). These bubbles may correspond to the secretion of saliva from the salivary canal into the dermis during probing.

#### Blood meal

**Figure 3:**
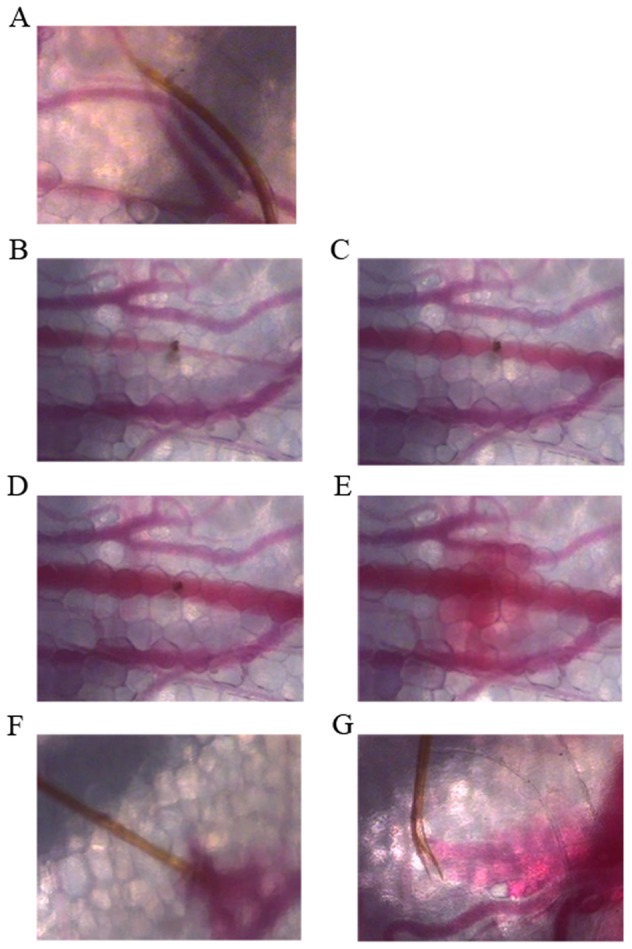
Intravital micrographs showing different blood meal types. A: The proboscis is inserted into the blood vessel and follows its lumen; B, C, D and E: the proboscis is inserted perpendicular to the blood vessel. The images were recorded at several time points after the start of blood feeding. The strength of suction decreases over time, as demonstrated by the gradual increase in blood vessel diameter until withdrawal of the proboscis; F and G: illustration of two types of pool feeding. In F, the proboscis is located in the pool of blood, whereas in G, the proboscis is sucking up blood from a blood pool some distance away. The proboscis is showed by an arrow.

Only 46.5% of the 200 mosquitoes observed were able to feed on blood, and 10% of those able to feed were unable to feed to repletion. Two types of feeding were observed: capillary feeding and pool feeding. In some cases, both types of feeding were observed (6.5%). Capillary feeding was observed the most frequently, and was itself of two types. The first type of capillary feeding involved the fascicle entering a blood vessel and following its lumen, remaining in the bloodstream (8% of blood meals). This type of behavior is clearly visible in Movie S5 and Figure 3A. At a higher magnification, blood from both parts of the blood vessel can clearly be seen flowing towards the labrum and red blood cells can be observed flushing up the food canal. In the second type of capillary feeding, which occurred more frequently, the fascicle penetrated the blood vessel at a right angle, with the tip of the labrum seeming to pierce the vessel wall (Movie S6). Blood sucking by the mosquito triggered a collapse of the vessel wall, due to the very strong suction, particularly at the start of the blood meal (Movie S7). Figures 3B to E illustrate this observation. The diameter of the vessel clearly decreases as a function of the intensity of suction exerted by the pharyngeal pump, which decreases towards the end of the blood meal. A large hemorrhage was observed at the end of feeding. Vessels of diverse sizes were selected for blood feeding. In Movie S8, a mosquito can be seen feeding on a blood vessel twice the size of the fascicle located close to a very large vessel. In Movie S9, the mosquito can be seen feeding in a large blood vessel.

Pool feeding was observed in 7.5% of the engorgements. In this case, a hemorrhage is triggered by the withdrawal of the proboscis from a blood vessel or the passage of the proboscis through a blood vessel. The insect detects the pool of blood and sucks it up, as shown in Movies S10 and S11. Figures 3F and G show two examples of pool feeding. In one case, the fascicle can be seen in the blood pool, whereas, in the other, the blood was sucked at some distance from the hemorrhage. However, pool feeding was never sufficient for complete repletion of the female. As soon as the source of blood had dried up, the insect began seeking another blood vessel to complete the blood meal by capillary feeding (5% of feedings).

Table 1 shows the influence of several physiological parameters of mice and mosquitoes on the various probing and feeding parameters. We studied a population of 127 mosquitoes, in two age groups (8 days and 23 days), unfed or blood fed on naive mice before observation. Most were eight days old (93%). The probing phase was observed in 83% of cases, with multiple probing (3 to 5) occurring in 91% of these cases. Probing began after 6.5 s and feeding began after 150 s. The median duration of probing was 142 s and the median duration of feeding was 240 s. Capillary feeding was the most frequent (59%). In most cases, the stylets were perpendicular (92%) to the vessel. Eight-day-old mosquitoes began to probe sooner (5 s vs. 32 s, p = 0.003), and had a shorter meal duration (133 s vs 366 s, p = 0.03).

**Table 1:** Influence of female mosquito's age on various parameters of probing and blood feeding observed using intravital microscopy.

N (%)	Anopheles		Total	P
	8 days-old	23 days-old	(n = 127)	
	(n = 118)	(n = 9)		
Start of probing (s)*	5 (1–19.5)	32 (18–43)	6.5 (1–21)	0.003
Probing duration (s)*	133 (73–258)	366 (220–399)	142 (73–258)	0.03
Capillary feeding (yes)	69 (61)	4 (44)	73 (59)	0.48
Pool feeding (yes)	7 (6)	0 (0)	7 (6)	0.99
Blood feeding duration (s)*	240 (150–329)	340 (270–368)	240 (156–345)	0.21
Capilllary feeding duration (s)*	205 (150–300)	340 (270–368)	225 (158–324)	0.15
Pool feeding duration (s)*	200 (60–300)	Missing	200 (60–300)	

* *Median (Q1–Q3).*

*All experiments were performed with an external temperature of 20°C+/−2°C.*

#### Effect of mosquito *Plasmodium* infection on blood feeding

Mosquitoes were infected with the NK65 strain of *Plasmodium berghei* expressing the GFP protein at the oocyst and sporozoite stages. We observed the behavior of infected mosquitoes either selected before blood feeding anesthetized on ice under a fluorescence microscope or after blood feeding with the same procedure to determine infection status. This second protocol overcame the need to subject the mosquitoes to the stress associated with selection at 4°C, but it was harder to ensure that there were equal numbers of infected and uninfected mosquitoes. We analyzed the behavior of both groups of mosquitoes (Table S1). We studied a population of 35 23-day-old mosquitoes, 26 of which were infected and nine of which were uninfected. Capillary feeding was most frequently observed (65%). All these mosquitoes inserted the proboscis vertically into an artery. There was no difference between infected and uninfected mosquitoes considering the probing and feeding times.

For the second protocol, for which no selection of infected mosquitoes was performed before blood meal, the time lag until the mosquito began probing and the duration of probing did not differ significantly between infected and uninfected mosquitoes (Table 2), but infected mosquitoes were more willing to probe and took longer to achieve repletion than uninfected mosquitoes.

**Table 2:** Role of *Plasmodium berghei* infection on probing and blood meal of *Anopheles gambiae* using the second protocol.

Infection status	probing	Number of females	time to probing (sec)	% of individual taking blood meal	probing time (sec)	Duration of blood meal (sec)
Infected	yes	19 (86%)*	39±51	73%	210±89	460±130*
	no	3				
Non infected	yes	4 (44%)*	54.6±95	75%	433±474	110±17*
	no	5				

In this experiment, the mosquitoes were not selected by their infective status prior to blood feeding observations but were checked for the presence of GFP-sporozoites thereafter.

* p<0.01.

#### Effect on blood feeding of immunized mice against mosquito saliva

**Figure 4:**
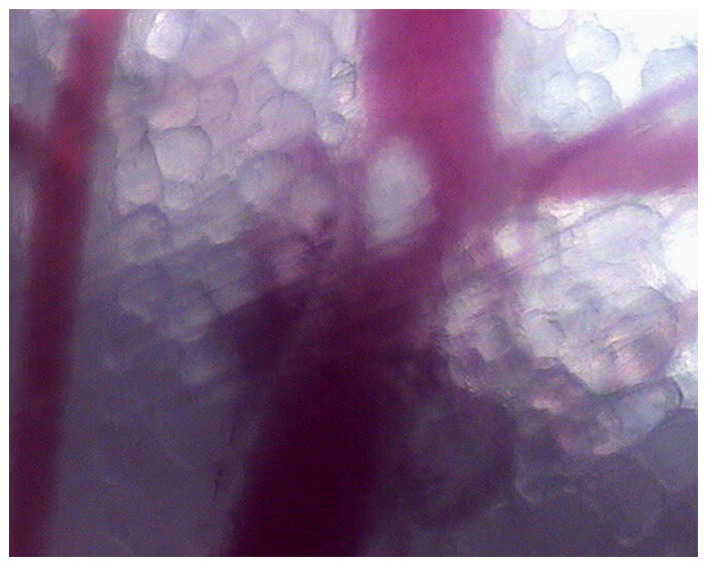
Intravital micrograph of a blood vessel after blood feeding. The mouse was immunized with saliva. A white area enlarged around the tip of the proboscis and remained visible after the withdrawal of the mouthparts.

For the purpose of the experiment, mice were subjected to various types of immunization. Some were bitten by unfed mosquitoes at various time points and the titer of anti-saliva antibodies was assessed by ELISA. Others were injected with rabbit anti-saliva IgG (200 µg for mice exposed to mosquitoes or 400 µg for naive mice), with or without prior active immunization with saliva. Those receiving bites and IgG were grouped in the “actively immunized” mice. We used intravital microscopy to record and measure the same parameters as above: probing time, the size of the blood vessels used for blood feeding and events within the blood vessel during engorgement (Table 3). We first compared the behavior of mosquitoes feeding on naive and saliva-immunized mice (active and passive immunizations). We studied 146 mosquitoes, 63 blood-fed on non-immune mice and 83 of which feeding on immunized mice. The results for the immunized and naive mice were significantly different, with a greater proportion of probing (84% vs 68%, p = 0.028), an earlier start to probing (5 s vs. 17 s, p = 0.001), a larger proportion of white areas in the vessel (53% vs 0%, p<0.001), a higher proportion of bites targeting the largest veins (57% vs 7%) and a greater median size of the largest vessels targeted (3 vs 2, p = 0.0096) for mosquitoes feeding on immunized mice. The proboscis was inserted at a right angle, the tip being clearly visible in the middle of the vessel. This observation differs from other observations of engorgement, in which the tip seemed to pierce the vessel wall. Once a white area had formed in immunized mice during feeding, it prevented blood from circulating in the vessel, as shown it Movie S12. The white area remained visible in the vessel for several minutes after the withdrawal of the proboscis (Figure 4).

**Table 3:** Comparison between mosquitoes feeding on saliva-immunized and non-immunized mice.

N (%)	Non-immunized mice	Immunized (active and passive) mice	Total	p
	(n = 63)	(n = 83)	(n = 146)	
*Anopheles* type				<0.001
- Non-infected unfed	28 (44)	83 (100)	111 (76)	
- Non-infected blood-fed	9 (14)	0 (0)	9 (6)	
- Infected	26 (41)	0 (0)	26 (18)	
8 day-old (yes)	28 (44)	83 (100)	111 (76)	<0.001
Probing (yes)	43 (68)	70 (84)	113 (77)	0.028
Time to start probing (sec)*	17 (8–32)	5 (1–18)	9 (1–23)	0.001
Time to start feeding (sec)*	240 (120–290)	144 (78–287)	174 (90–290)	0.087
Probing duration (sec)*	224 (113–288)	133 (73–242)	158 (77–262)	0.11
Capillary feeding (yes)	29 (60)	52 (66)	81 (64)	0.57
Pool feeding (yes)	3 (6)	4 (5)	7 (6)	0.99
Blood meal duration (sec)*	300 (185–420)	240 (150–300)	240 (180–385)	0.11
Capillary feeding duration (sec)*	290 (180–393)	240 (165–317)	240 (180–350)	0.36
Perpendical position of fascicule (yes)	21 (91)	39 (93)	60 (92)	0.99
White area (yes)	0 (0)	20 (53)	20 (35)	<0.001
Size of blood vessel*	2 (1–3)	3 (2–4)	2.75 (1.3–4)	0.0096

* *Median (Q1–Q3).*

We then compared the different types of immunization (Table 4). We studied 7 immunized mice: 4 immunized passively (32 mosquitoes were fed on these mice) and 3 immunized actively and passively (51 mosquitoes were fed on these mice), which were grouped together since no significant difference was found between actively immunized and mice immunized actively prior receiving an injection of 200 µg of anti-saliva antibodies. Significant differences were observed between the different types of immunization. The proportion of mosquitoes willing to probe was higher in mice that had been immunized passively (100% vs 75%, p = 0.001) and probing began earlier in these mice (1 second vs 11 seconds, p<0.001). Capillary feeding was more frequent in mice subjected to passive immunization (91% vs 49%, p<0.001). One factor was independently associated to passive immunization, namely probing start time fewer than 5 s (OR = 36.4 [95%CI: 8.8–150]).

**Table 4:** Comparison between the various types of immunization.

N (%)	Passive	Active	Total	p
	(n = 32)	(n = 51)	(n = 83)	
Probing	32 (100)	38 (75)	70 (84)	0.001
Probing start time (s)*	1 (1-1)	11 (5–26)	5 (1–18)	<0.001
Blood feeding start time (s)*	120 (70–300)	172 (112–266)	144 (78–287)	0.50
Duration of probing (s)*	109 (70–300)	148 (80–235)	133 (73–242)	0.80
Capillary feeding	29 (91)	23 (49)	52 (66)	<0.001
Pool feeding	2 (6)	2 (4)	4 (5)	0.99
Blood feeding duration (s)*	240 (180–339)	190 (120–300)	240 (150–300)	0.12
Capillary feeding duration (s)*	240 (180–386)	190 (145–300)	240 (165–317)	0.078
Perpendicular position of the proboscis	20 (95)	19 (90)	39 (93)	0.99
White area	11 (55)	9 (50)	20 (53)	0.76
Vessel size*	3.35 (1.3–4.6)	3 (2–4)	3 (2–4)	0.93

* *Median (Q1–Q3).*

#### Imaging sporozoite release in the skin

**Figure 5:**
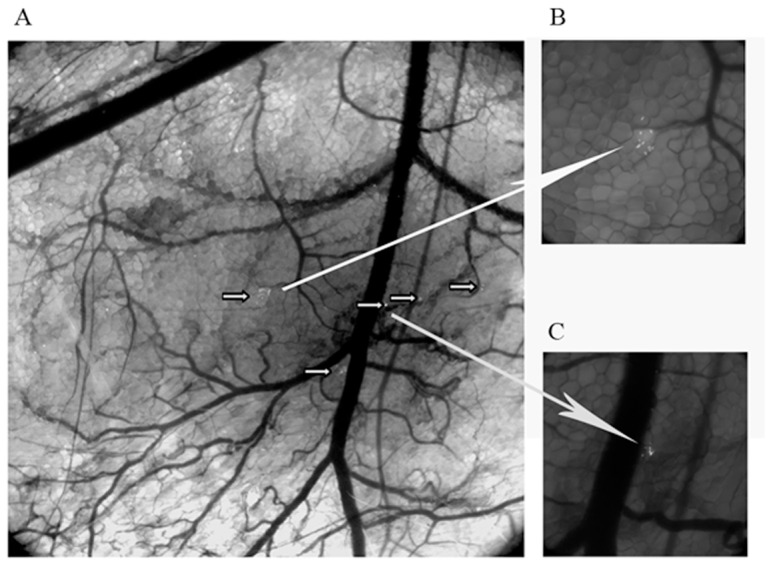
Intravital fluorescence micrograph showing several deposits of sporozoites in the mouse skin. A: Global view of mouse skin bitten by a mosquito infected with GFP-labeled sporozoites. Small arrows indicate sporozoite deposits; B and C: enlargement of two pools of sporozoites indicated by large arrows.

As the mosquitoes were infected with a strain of *Plasmodium berghei* that expresses GFP at the oocyst and sporozoite stages, we were able to capture images of sporozoite release in the dermis of the back of shaved anesthetized mice with a stereoscopic fluorescence microscope. We were also able to observe the behavior of the sporozoites over short periods of time in live animals. Several pools of sporozoites were identified at various sites within the dermis (Figure 5A). Several were located close to blood vessels, as indicated by the arrows in Figure 5A. A magnification of two zones to which sporozoites were delivered is shown in Figures 5B and 5C. We focused on a single cluster of sporozoites and recorded their movement over a period of 15 minutes. As shown on Movie S13, 10 sporozoites were clearly visible on the lower part of the dermis, very close to the fat cells. We occasionally observed sporozoites in the upper part of the dermis. They moved more rapidly, with one sporozoite seeming to be carried away in the bloodstream.

### Visualizing the effects of mosquito bites on the skin

**Figure 6:**
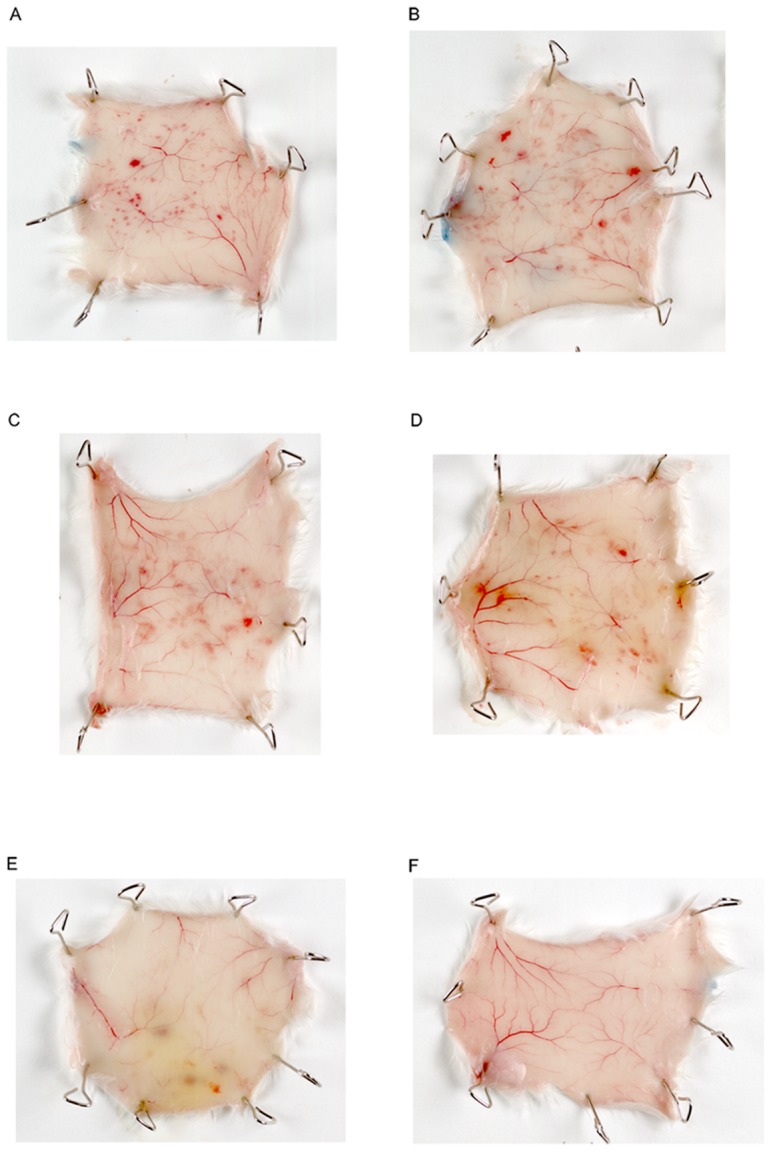
Local tissue damage on the inner surface of the skin after uninfected mosquito bites. Mice were exposed to the bites of 50 mosquitoes for 15 minutes and were killed at various time points: A) 1 h; B) 3 h; C) 5 h; D) 8 h; E) 24 h; F) control. Thirty minutes before the mice were killed, they were injected with Evans Blue, for the monitoring of capillary extravasation.

**Figure 7:**
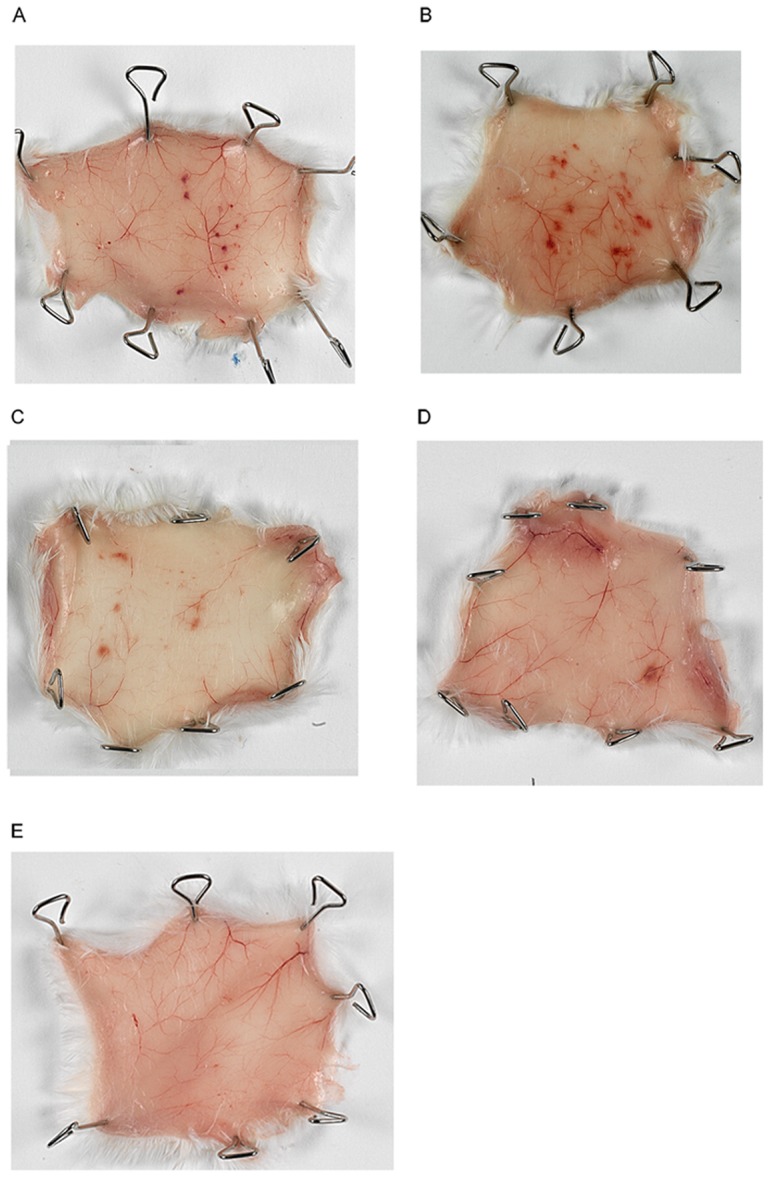
Local tissue damage on the inner surface of the skin after *Plasmodium berghei*-infected mosquito bites. Infected mosquitoes were selected by the visualization of fluorescent sporozoites in the salivary glands of cold-anesthetized insects under a fluorescence microscope. Mice were exposed to the bites of 50 infected mosquitoes for 15 minutes and killed at various time points: A) 1 h; B) 3 h; C) 16 h; D) 24 h; E) control. Thirty minutes before the mice were killed, they were injected with Evans Blue for the monitoring of capillary extravasation.

Mosquito bites usually cause local cutaneous inflammatory reactions, with small papules, erythema and pruritic swelling. We followed the kinetics of the local reaction in naive mice bitten by 50 uninfected mosquitoes and sacrificed at various time points, 30 minutes after the injection of Evans Blue. No Evans Blue was injected for histological examinations. Saliva induced a strong inflammatory response in naive mice (Figure 6). Vasodilation of the blood vessels was observed until 8 hours after feeding. Bleeding at the site of the bite, together with capillary extravasation, as demonstrated by blue staining with Evans Blue, was the first sign of inflammation. Hemorrhages were punctuate at 1 h, and tended to enlarge over the following two hours. Between 5 h and 8 h, the outline of the hemorrhages became blurred, and the bleeding had almost disappeared by 24 h. Edema was also observed until 8 h after the bite, decreasing by 48 h (data not shown). We performed the same experiment with *Plasmodium berghei*-infected mosquitoes selected on the basis of GFP fluorescence under the microscope before contact with mice (Figure 7). The mice were exposed to similar numbers of infected and uninfected mosquitoes, but fewer of the infected mosquitoes were able to bite. Extravascular permeabilization, as assessed after Evans Blue injection, was not detected. Vascular congestion peaked 3 h after the bite. The extent of the hemorrhages was similar to that after uninfected mosquito bites.

Histological investigations were performed on lesions resulting from the feeding of uninfected or *Plasmodium berghei*-infected mosquitoes. Serial sections of the same skin were stained with hematoxylin-eosin or Giemsa stain or were subjected to immunohistochemistry (IHC) with antibodies directed against saliva components, thereby allowing a comparison of the various staining at the same regions for the same time points.

**Figure 8:**
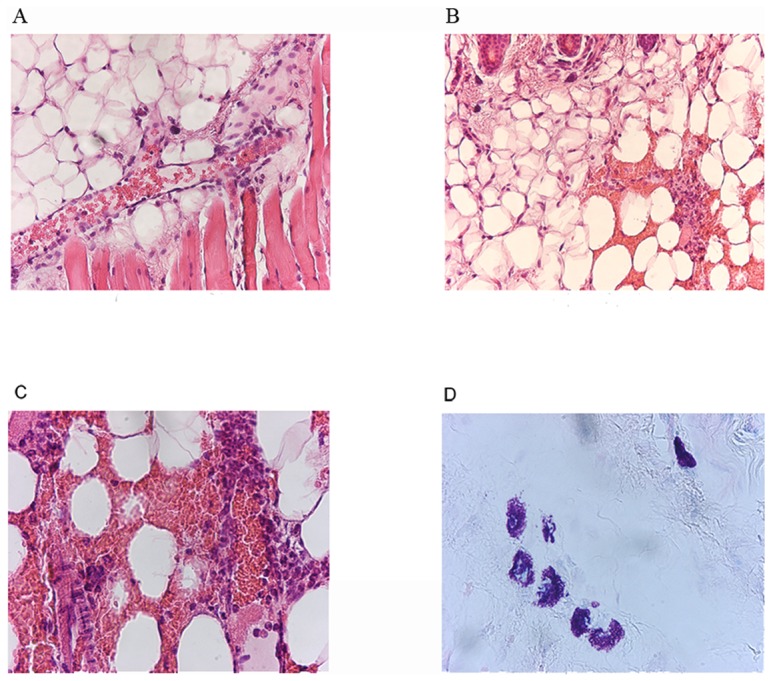
Photomicrographs of skin sections and underlying tissues of naive mice bitten by uninfected *Anopheles gambiae* mosquitoes. These observations were made on mice killed one hour after the bite. A, B, C: HE staining; D: Giemsa staining; B. The magnification was ×20 for A and B and ×40 for C and D.

Areas of bleeding were observed one hour after the bite (Figure 8). The inflammatory response originated in blood vessels, with an initial stage characterized by polynuclear margination (Figure 8A). At this early time point, polynuclear cells had already started to invade the dermis. In Figures 8B and C, large infiltrates of polynuclear cells can be seen in the dermis, together with huge areas of red blood cells. Giemsa staining showed that some of the mast cells of the lesion site were degranulated. Three hours after the bite, a large number of mast cells were present in the vicinity of the infiltrate, and all were degranulated. Some were still degranulated 24 h after the bite, and the infiltrates of polynuclear cells remained visible (data not shown). Mast cells were present, but none were degranulated, at this time point. We investigated whether the degranulation of mast cells one hour after the bite was linked to the activation of other cells or was a primary effect triggered by saliva, by carrying out similar observations at earlier time points. Mice were exposed to mosquito bites and sacrificed at evenly spaced time points between five and 30 minutes. Mast cell degranulation was observed in the areas of hemorrhage within five minutes of the bite (data not shown).

**Figure 9:**
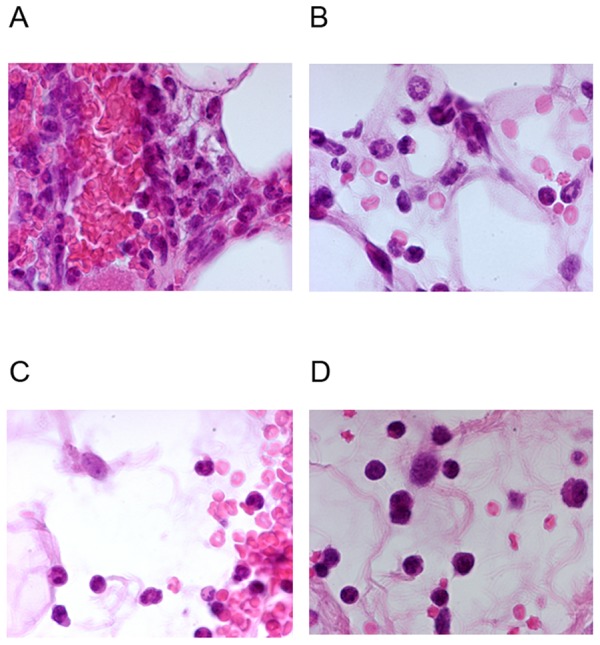
Observation of areas of hemorrhage at high magnification. Skin sections from mice killed at the times indicated were stained with HE 1 h (A), 3 h (B), 8 h (C) and 24 h (D) after the bite. Magnification ×100.

We observed areas of inflammation of HE-stained tissues at a higher magnification (Figure 9). One hour after the bite, macrophages and polynuclear cells were observed in the vicinity of red blood cells. The red blood cells were still intact. Three hours after the bite, neutrophils, eosinophils and macrophages were visible among the red blood cells, which were either intact or shriveled. A few lymphocytes were observed eight hours after the bite, and the number of these cells had increased 24 hours after the bite (data not shown).

**Figure 10:**
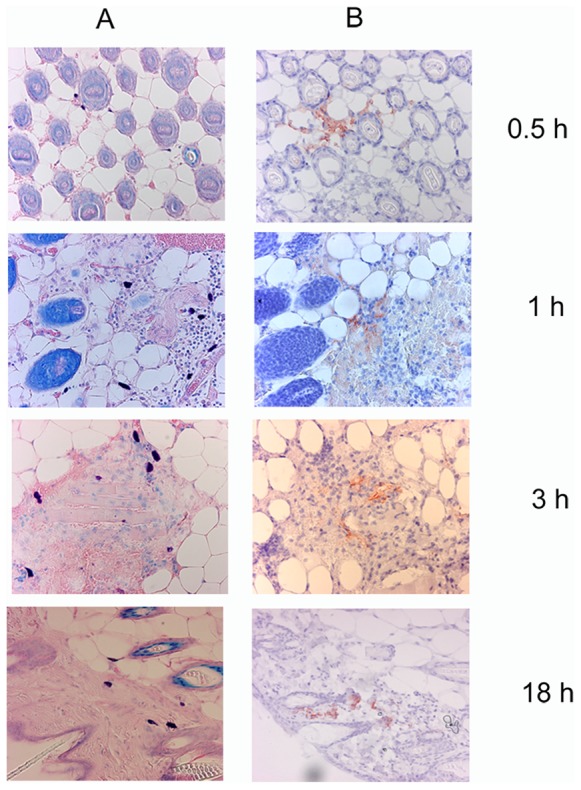
Localization of saliva in the dermis of mice bitten by *Anopheles gambiae*. Saliva was detected with rabbit anti-saliva antibodies 30 minutes, 1 h, 3 h and 18 h after the bite. A: Giemsa staining, B: saliva staining. Magnification: ×20.

**Figure 11:**
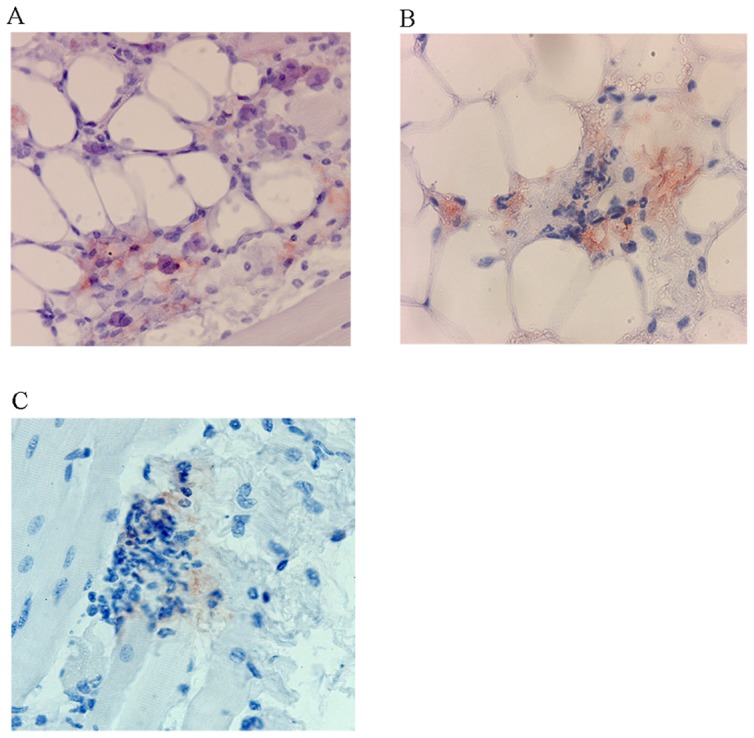
Localization of polynuclear cells and saliva in the dermis. Saliva was detected with rabbit anti-saliva antibodies A) 30 minutes, B) 1 h, and C) 3 h Saliva was stained with rabbit anti-saliva antibodies. Magnification: ×40.

We monitored the location of saliva after the bite by immunohistochemical staining with anti-saliva antibodies of the same portions of skin previously observed after HE or Giemsa stainings. Saliva deposits were detected in the lower dermis as early as 15 minutes after the bite and were clearly detectable until eight hours after the bite. These deposits were no longer detected 24 hours after the bite. The location of the saliva varied over time. From 30 minutes to one hour after the bite, saliva was detected in the lower dermis, close to large blood vessels or dispersed throughout the tissue (Figure 10), presumably due to several releases of saliva from the salivary channel during different attempts to probe the skin area. Three hours after the bite, saliva deposits were observed close to or within hair follicles. This distribution was even more marked eight hours after the bite. Saliva deposits were observed until 18 hours after the bite (Figure 10). Clusters of cells, mostly polynuclear, were colocalized with saliva deposits (Figure 11). Giemsa staining of the same areas revealed the presence of degranulated mast cells in the vicinity of the saliva deposits (data not shown).

**Figure 12:**
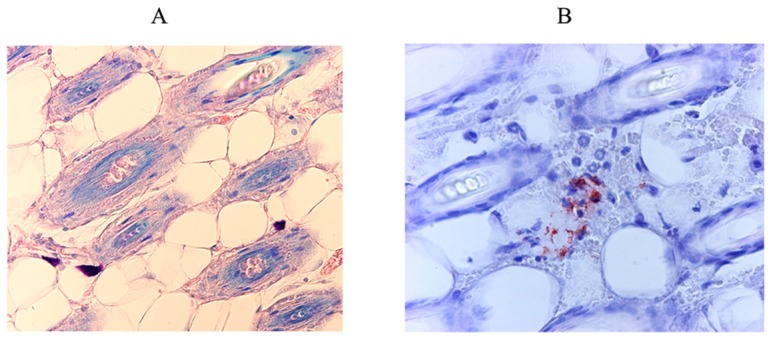
Localization of mast cells (A) and sporozoites (B) on skin sections from mice bitten by *Plasmodium berghei*-infected mosquitoes. Mast cells were localized by Giemsa staining 1 h after bite. Sporozoites were stained with a monoclonal anti-CS antibody. Magnification: ×40.

We compared the Giemsa and anti-CSP staining patterns of the same area of skin at different time point. One hour after the bite, mast cells were observed in the vicinity of sporozoites (Figure 12). Fewer polynuclear cells were associated with the saliva deposits than for uninfected saliva.

**Figure 13:**
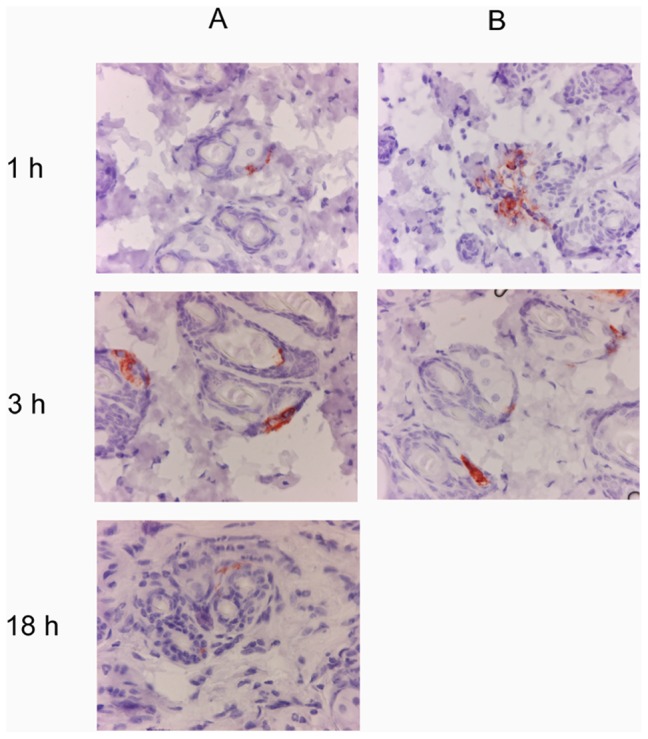
Detection of sporozoites (A) and saliva (B) deposits in the dermis after *Plasmodium berghei*-infected mosquito bites. Sporozoites were identified with a monoclonal anti-CSP antibody and saliva was characterized with rabbit anti-saliva antibodies. Magnification: ×40.

We then compared the anti-CSP staining and anti-saliva staining patterns of a given skin area at various times after the bite (Figure 13). Sporozoites were detected until 18 h after the bite. They were colocalized with saliva 30 minutes, 1 and 3 h after the bite, initially within the dermis and then within hair follicles.

## Discussion

Previously reported observations of mosquito bites have mostly concerned bites to frog legs or mouse ears. We used a less specialized model than the ear, which contains cartilage and has a thin skin unlike that covering the rest of the body. We also felt that the observation of *Anopheles gambiae* mosquitoes biting a mammalian host would be the most appropriate model for obtaining useful data concerning biting and parasite interactions. Our observations are supported by video recordings, still photographs and sections through the tissues of bitten mice. They enabled us to describe in more detail the interaction between *Anopheles gambiae* and the skin of naive or saliva-sensitized mice. We also used mosquitoes infected with *Plasmodium berghei* and various tools, including antibodies and fluorescent strains, to study parasite transmission.

Videos of the movement of the mosquitoes' mouthparts within the skin revealed that the tip of the labrum was highly flexible during the probing phase. A large area under the skin was probed, without the mosquito having to withdraw its proboscis or change the point of entry. Similar observations were previously reported by [14] for *Aedes aegypti* feeding on the webbed skin of frogs. The labral elevator and retractor muscles probably play a major role in this flexibility. Interestingly, our observations also suggest that the localization of blood vessels by mosquitoes may be fortuitous. Despite the use of a different experimental set-up and model animal, Gordon and Lumsden [14] also concluded that chance played a major role in blood detection. However, our experiments were performed with laboratory-reared insects and the rapid location of blood vessels is a trait known not to be maintained in the laboratory [20], [21]

We recorded the blood feeding phase. Young mosquitoes fed more rapidly than older mosquitoes and *Anopheles gambiae* was found to behave essentially as a capillary feeding insect to achieve repletion. Blood feeding by mosquitoes to repletion was one important aspect in the escape of larvae for *W. bancrofti* transmission [22]. Pool feeding was observed on some occasions [23] but was not efficient and did not result in repletion. These observations contrast with those for blood feeding by *Aedes aegypti*, which can become fully engorged after pool feeding [14].

Parasitization has been reported to change insect behavior [24]. We found that *Plasmodium berghei*-infected insects were more willing to probe than uninfected insects. This observation is consistent with the results of Anderson *et al.* [25], who found that *Plasmodium*-infected mosquitoes with sporozoites in their salivary glands were less likely to give up their attempt to feed than uninfected mosquitoes. This change in insect behavior, with a larger number of probing attempts, may be accounted for by changes in the expression of salivary gland components [19], [26] or a modulation of the nervous system by the parasite [27]. However, we observed no difference in probing time between *Plasmodium berghei*-infected and uninfected *A. gambiae* in this study. This observation is consistent with the findings of Pumpuni *et al.* [28], who found no difference in total feeding time between *Plasmodium yoelli*-infected and uninfected *Anopheles stephensi*.

The ability of mammals to produce antibodies against mosquito saliva antigens is well established [29], [30], [31]. We therefore investigated whether the presence of antibodies in the blood of animals interfered with the blood meal. We found that immunized mice were more attractive to mosquitoes than non immunized mice and that the mosquitoes probed the immunized mice more rapidly. For feeding the first step in the behavioural sequence is to sample the substrate and determine if the food is suitable. This happens if the appropriate feeding stimulus, a phagostimulant, is present. Presence or absence of the phagostimulant results in either feeding or not, and in its absence the insect moves on continuing to look for food [32]. showed an enhanced feeding success of *Aedes aegypti* mosquitoes on parasitemic hosts. In our case, it seems that the presence of anti-saliva antibodies could act indirectly as a phagostimulant. In mosquito sensitized against saliva, we can expect a modification of the skin by the interaction of saliva with the innate immune system [4] [33], resulting in a pro-inflammatory host response causing a vasodilatation of blood vessels. In mice passively immunized, we propose that antibodies that have been passively transferred may modify the site of bite as shown by [23], [34] Interestingly, the biting mosquitoes tended to carry out capillary feeding in significantly larger blood vessels in immunized than in naive mice. This observation may be explained because of the vasodilatation due to the pro-inflammatory response induced in saliva-sensitized mice. It could also be explained by the presence of anti-saliva antibodies in smaller vessels that may completely inhibit blood-feeding. This observation has potential implications, because the concentration of parasite stages infectious for mosquitoes may vary with vessel size. The concentration of gametocytes is higher in capillaries, whereas the percentage of old trophozoites is higher in larger vessels [35]. By contrast, microfilarial density in small peripheral blood vessels has been shown to be lower than that in large blood vessels [36].

A white area developed in direct contact with the proboscis of mosquitoes feeding on salivary-sensitized animals or mice injected with anti-saliva antibodies. Precipitating antibodies have been found in the blood of guinea pigs or rabbits bitten by *Aedes aegypti* [37]. The white area may therefore correspond to an immune reaction to saliva delivered to the bloodstream during blood feeding. This assumes that mosquitoes salivate during blood feeding, resulting in the exposure of saliva antigens to blood components. This hypothesis is entirely tenable, because Griffiths and Gordon [38] observed mosquito salivation within a blood vessel during blood feeding and Kebaier *et al.* [34] reported an apparent precipitant reaction at the distal end of the mosquito proboscis during intravital microscopy of infected mosquitoes feeding on mice previously passively immunized with antibodies against *Plasmodium berghei*.

In recent years, evidence has accumulated that skin cells not only provide a physical barrier between the body and the environment, but also actively modulate both innate and adaptive immune responses, by producing and responding to various cytokines and chemokines upon stimulation [39]. The physical barrier is breached during arthropod feeding, and the release of saliva has been shown to modulate immune responses [6]. We therefore followed the time-course of the local reaction in naive animals, to identify the cells involved in this process, and to investigate the role played by saliva. The skin of naive animals bitten by mosquitoes was characterized by the presence of hemorrhages, vasodilated blood vessels and an infiltrating edema, all of which are typically observed during intense inflammatory reactions. Saliva in the skin was visualized for the first time in this study by immunohistochemistry (IHC) with anti-saliva antibodies. Saliva deposits remained in the dermis for a long period of time after the bite, in large areas probed by the mosquitoes, and clusters of mostly polynuclear and mast cells were found either at or close to the site of the deposits. Finally, saliva was found concentrated in hair follicles. According to the video microscopy images, hemorrhages resulted either from the proboscis damaging a blood vessel during probing or from the withdrawal of the mosquito's mouthparts from the blood vessel at the end of the feeding phase. The formation of skin lesions during the probing phase is detrimental to the vector, because such lesions may lead to its discovery. However, pain and itch sensations are not observed during mosquito feeding. These reactions seemed to peak one to three hours after the bite, consistent with the observations of Demeure *et al.* [33]. We found that mast cells began to degranulate as little as five minutes after the bite. Mast cells are known to play an important role in immediate hypersensitivity reactions and inflammation (for a review, see [40]). Mast cell mediators have diverse biological activities, including neutrophil and eosinophil chemotaxis. Histamine release is triggered by IgE binding to Fc receptors or. As the mice were not previously sensitized to saliva, the action of histamine-releasing factors may explain our observations. Trancriptome and proteome studies of *A. gambiae* salivary glands have shown the presence of TCTP (translationaly controlled tumor protein), which could potentially act as a histamine-releasing factor [16], to be present in these organs. We observed that saliva deposits in the skin were associated with polynuclear cells. Owhashi *et al.* [41], [42] showed that the saliva of anopheline mosquitoes contains factors that are chemotactic for host neutrophils (NCF). Moreover, a protein of the chitinase family has been shown to attract eosinophils in *Anopheles* saliva [41]. Anti-inflammatory proteins, including molecules from the D7 family and apyrase, have also been identified in *Anopheles* saliva [43], [44]. The presence of compounds with opposite effects raises questions about the role of these compounds in blood-feeding and parasite transmission. Vasodilation and the increase in vascular permeability induced by proinflammatory molecules may decrease the duration of blood feeding. Conversely, they might also attract the host's attention to the bite, potentially resulting in the death of the arthropod. The action of proinflammatory molecules is undoubtedly counterbalanced, at least during blood feeding, by that of anti-inflammatory molecules. Mice lacking histamine receptors have been shown to be more resistant to *Plasmodium* infection [45], which suggests that inflammation interferes with parasite transmission. The presence of the parasite may also modify the expression of salivary components. For example, apyrase, an anti-inflammatory molecule, has been reported to be less abundant in the salivary glands of infected mosquitoes than in those of uninfected mosquitoes [19], [26].

We then considered the fate of sporozoites after their deposition in the skin with the saliva. The release of fluorescent sporozoites was visualized in the dermis. They had been injected into non vascular tissues at various sites in the skin, as previously shown [23]. Only a few displayed clear forward-gliding locomotion, the others seeming to remain in their initial position. This observation is consistent with the findings of Amino *et al.* [46], who observed that sporozoites delivered to the ear displayed robust forward gliding but that most remained in the image volume over time. [47] (1997) indicated that first sporozoites left the skin to invade the blood around 15 min after injection. Vanderberg and Frevert [23] showed that substantially more sporozoites take a significant longer time to enter the blood. IHC showed that CSP could still be identified in the skin 18 h after injection, consistent with the findings of Yamauchi *et al.*, [48], who detected sporozoites in the skin by PCR until 42 h after injection, and those of Gueirard *et al.* [49] who detected sporozoites in hair follicles over a period of several weeks. Our results also suggest that saliva from sporozoite-infected salivary glands may stimulate inflammation less strongly than saliva from uninfected salivary glands. This observation is consistent with our previous finding that levels of homologs of anophensin, the kallikrein-kinin system inhibitor from the salivary gland of *Anopheles stephensi* [50], are higher in infected salivary glands than in uninfected glands (unpublished results). The presence of *Plasmodium* in the salivary glands and salivary canal may also decrease the amount of saliva injected into the vertebrate, as shown in *Wolbachia*-infected *Aedes aegypti* [51], [52].

In conclusion, this study provides original movies of the various phases of blood feeding by young and older parasite-infected and uninfected females, on naive and saliva-sensitized mice. The duration of probing phase increased with the age of the mosquito, but was not influenced by any of the other factors. We also demonstrated that the behavior of *Plasmodium*-infected mosquitoes was modified in a way that might increase pathogen transmission.

Histological observations of skin sections from mice bitten by uninfected and *Plasmodium*-infected mosquitoes provided the first demonstration of the colocalization of mosquito saliva with parasites in various skin compartments over time. Eight hours after the bite, saliva, which has immunosuppressive activity, was found to be associated with sporozoites in a compartment that has itself also been described as immunosuppressive [53]. These observations suggest that the survival of parasites in the skin may be influenced by salivary components present in the same compartment. Some saliva proteins may bind to these parasites, protecting them and directing them to this particular environment, in which they can survive for weeks. Moreover, the immunosuppressive effects of mosquito saliva may act in synergy with the suppressive immunomodulatory mechanisms induced by filariae [54]. Co-infection with other parasites is common in humans with filariasis, which may modulate protective immune responses to malaria [55]. Studies of the interplay between vectors, pathogens and their transmission through saliva therefore constitute an interesting approach to the design of new approaches to blocking pathogen transmission.

## Supporting Information

Figure S1 **Equipment used for real time blood feeding examination.** A Nikon Eclipse TE200 reverse microscope was connected to a digital color video camera.
(TIF)

Figure S2 *Anopheles gambiae* during blood feeding (A) and *Anopheles gambiae* after blood feeding (B).
(TIF)

Movie S1 **Mouthparts of** ***Anopheles gambiae*** **moving under the skin.**
(DIVX)

Movie S2 **Mouthparts of** ***Anopheles gambiae*** **moving under the skin.**
(MPG)

Movie S3 **Local tissue damage triggered by** ***Anopheles gambiae*** **probing.**
(MOV)

Movie S4 **Putative salivation by** ***Anopheles gambiae*** **during the probing phase.**
(MOV)

Movie S5 **Capillary feeding by** ***Anopheles gambiae*** **.** The proboscis is inserted along the lumen of the blood vessel.
(DIVX)

Movie S6 **Capillary feeding by** ***Anopheles gambiae*** **.** The proboscis is inserted into the blood vessel at a right angle.
(DIVX)

Movie S7 **Illustration of the strength of blood sucking.**
(DIVX)

Movie S8 **Mosquito blood feeding in a medium size blood vessel.**
(DIVX)

Movie S9 **Mosquito blood feeding in a large blood vessel.**
(DIVX)

Movie S10 ***Anopheles gambiae*** **pool feeding.**
(MOV)

Movie S11 **Mosquito feeding on a mouse immunized against saliva.** Beginning of engorgement and formation of a white area around the proboscis.
(DIVX)

Movie S12 **Mosquito feeding on a mouse immunized against saliva.** The mosquito has withdrawn its mouthparts and the white area remains for several seconds in the blood vessel
(MOV)

Movie S13 **Recording of sporozoite movement in the skin of a mouse.** The sporozoites can be identified on the basis of their GFP fluorescence. Their movements were recorded over a period of 15 minutes.
(MOV)

Table S1 **Comparison between infected and non-infected 23 day-old female mosquitoes**
(DOC)

## References

[1] Burkot TR, Molineaux L, Graves PM, Paru R, Battistutta D, et al (1990) The prevalence of naturally acquired multiple infections of Wuchereria bancrofti and human malarias in anophelines. Parasitology 100 Pt 3: 369–375. 2194153 10.1017/s003118200007863x

[2] Muturi EJ, Jacob BG, Kim CH, Mbogo CM, Novak RJ (2008) Are coinfections of malaria and filariasis of any epidemiological significance? Parasitol Res 102: 175–181. 18026992 10.1007/s00436-007-0779-1

[3] Ribeiro JM (1995) Blood-feeding arthropods: live syringes or invertebrate pharmacologists? Infect Agents Dis 4: 143–152. 8548192

[4] Ribeiro JM, Francischetti IM (2003) Role of arthropod saliva in blood feeding: sialome and post-sialome perspectives. Annu Rev Entomol 48: 73–88. 12194906 10.1146/annurev.ento.48.060402.102812

[5] Donovan MJ, Messmore AS, Scrafford DA, Sacks DL, Kamhawi S, et al (2007) Uninfected mosquito bites confer protection against infection with malaria parasites. Infect Immun 75: 2523–2530. 17339356 10.1128/IAI.01928-06 PMC1865743

[6] Schneider BS, Higgs S (2008) The enhancement of arbovirus transmission and disease by mosquito saliva is associated with modulation of the host immune response. Trans R Soc Trop Med Hyg 102: 400–408. 18342898 10.1016/j.trstmh.2008.01.024 PMC2561286

[7] Githeko AK, Adungo NI, Karanja DM, Hawley WA, Vulule JM, et al (1996) Some observations on the biting behavior of Anopheles gambiae s.s., Anopheles arabiensis, and Anopheles funestus and their implications for malaria control. Exp Parasitol 82: 306–315. 8631382 10.1006/expr.1996.0038

[8] Mahande A, Mosha F, Mahande J, Kweka E (2007) Feeding and resting behaviour of malaria vector, Anopheles arabiensis with reference to zooprophylaxis. Malar J 6: 100. 17663787 10.1186/1475-2875-6-100 PMC1964787

[9] Qiu YT, Smallegange RC, JJ VANL, Takken W (2011) Behavioural responses of Anopheles gambiae sensu stricto to components of human breath, sweat and urine depend on mixture composition and concentration. Med Vet Entomol 25: 247–255. 21108650 10.1111/j.1365-2915.2010.00924.x

[10] Rossignol PA, Ribeiro JM, Spielman A (1986) Increased biting rate and reduced fertility in sporozoite-infected mosquitoes. Am J Trop Med Hyg 35: 277–279. 3953943 10.4269/ajtmh.1986.35.277

[11] Wekesa JW, Copeland RS, Mwangi RW (1992) Effect of Plasmodium falciparum on blood feeding behavior of naturally infected Anopheles mosquitoes in western Kenya. Am J Trop Med Hyg 47: 484–488. 1443347 10.4269/ajtmh.1992.47.484

[12] Garcia ES, Mello CB, Azambuja P, Ribeiro JM (1994) Rhodnius prolixus: salivary antihemostatic components decrease with Trypanosoma rangeli infection. Exp Parasitol 78: 287–293. 8162960 10.1006/expr.1994.1030

[13] Koella JC, Sorensen FL, Anderson RA (1998) The malaria parasite, Plasmodium falciparum, increases the frequency of multiple feeding of its mosquito vector, Anopheles gambiae. Proc Biol Sci 265: 763–768. 9628035 10.1098/rspb.1998.0358 PMC1689045

[14] Gordon RM, Lumsden WHR (1939) A study of the behaviour of the pouth-parts of mosquitoes when taking up blood from living tissue; together with some observations on the ingestion of microfilariae. Ann trop Med Parasit 33: 259–278.

[15] Robinson GG (1939) The mouthparts and their function in the female mosquito, Anopheles maculipennis. Parasitology 31: 212–242.

[16] Rosinski-Chupin I, Briolay J, Brouilly P, Perrot S, Gomez SM, et al (2007) SAGE analysis of mosquito salivary gland transcriptomes during Plasmodium invasion. Cell Microbiol 9: 708–724. 17054438 10.1111/j.1462-5822.2006.00822.x

[17] Petit G (1985) Ingestion des hématozoaires par le vecteur. Analyse de 4 filaires parasites d'un Saïmiri. Ann Parasitol Hum Comp 60: 247–297. 10.1051/parasite/1985604455 4083676

[18] Natarajan R, Thathy V, Mota MM, Hafalla JC, Menard R, et al (2001) Fluorescent Plasmodium berghei sporozoites and pre-erythrocytic stages: a new tool to study mosquito and mammalian host interactions with malaria parasites. Cell Microbiol 3: 371–379. 11422080 10.1046/j.1462-5822.2001.00117.x

[19] Choumet V, Carmi-Leroy A, Laurent C, Lenormand P, Rousselle JC, et al (2007) The salivary glands and saliva of Anopheles gambiae as an essential step in the Plasmodium life cycle: a global proteomic study. Proteomics 7: 3384–3394. 17849406 10.1002/pmic.200700334

[20] Chadee DD, Beier JC (1997) Factors influencing the duration of blood-feeding by laboratory-reared and wild Aedes aegypti (Diptera: Culicidae) from Trinidad, West Indies. Ann Trop Med Parasitol 91: 199–207. 9307662 10.1080/00034983.1997.11813130

[21] Chadee DD, Beier JC, Mohammed RT (2002) Fast and slow blood-feeding durations of Aedes aegypti mosquitoes in Trinidad. J Vector Ecol 27: 172–177. 12546453

[22] Lardeux F, Cheffort J (1996) Behavior of Wuchereria bancrofti (Filariidea: Onchocercidae) infective larvae in the vector Aedes polynesiensis (Diptera:Culicidae) in relation to parasite transmission. J Med Entomol 33: 516–524. 8699443 10.1093/jmedent/33.4.516

[23] Vanderberg JP, Frevert U (2004) Intravital microscopy demonstrating antibody-mediated immobilisation of Plasmodium berghei sporozoites injected into skin by mosquitoes. Int J Parasitol 34: 991–996. 15313126 10.1016/j.ijpara.2004.05.005

[24] Hurd H (2003) Manipulation of medically important insect vectors by their parasites. Annu Rev Entomol 48: 141–161. 12414739 10.1146/annurev.ento.48.091801.112722

[25] Anderson RA, Koella JC, Hurd H (1999) The effect of Plasmodium yoelii nigeriensis infection on the feeding persistence of Anopheles stephensi Liston throughout the sporogonic cycle. Proc Biol Sci 266: 1729–1733. 10518321 10.1098/rspb.1999.0839 PMC1690202

[26] Rossignol PA, Ribeiro JM, Spielman A (1984) Increased intradermal probing time in sporozoite-infected mosquitoes. Am J Trop Med Hyg 33: 17–20. 6696175 10.4269/ajtmh.1984.33.17

[27] Lefevre T, Thomas F, Schwartz A, Levashina E, Blandin S, et al (2007) Malaria Plasmodium agent induces alteration in the head proteome of their Anopheles mosquito host. Proteomics 7: 1908–1915. 17464940 10.1002/pmic.200601021

[28] Pumpuni CB, Mendis C, Beier JC (1997) Plasmodium yoelii sporozoite infectivity varies as a function of sporozoite loads in Anopheles stephensi mosquitoes. J Parasitol 83: 652–655. 9267407

[29] Waitayakul A, Somsri S, Sattabongkot J, Looareesuwan S, Cui L, et al (2006) Natural human humoral response to salivary gland proteins of Anopheles mosquitoes in Thailand. Acta Trop 98: 66–73. 16530153 10.1016/j.actatropica.2006.02.004

[30] Remoue F, Cisse B, Ba F, Sokhna C, Herve JP, et al (2006) Evaluation of the antibody response to Anopheles salivary antigens as a potential marker of risk of malaria. Trans R Soc Trop Med Hyg 100: 363–370. 16310235 10.1016/j.trstmh.2005.06.032

[31] Orlandi-Pradines E, Almeras L, Denis de Senneville L, Barbe S, Remoue F, et al (2007) Antibody response against saliva antigens of Anopheles gambiae and Aedes aegypti in travellers in tropical Africa. Microbes Infect 9: 1454–1462. 17913537 10.1016/j.micinf.2007.07.012

[32] Rossignol PA, Ribeiro JM, Jungery M, Turell MJ, Spielman A, et al (1985) Enhanced mosquito blood-finding success on parasitemic hosts: evidence for vector-parasite mutualism. Proc Natl Acad Sci U S A 82: 7725–7727. 3865192 10.1073/pnas.82.22.7725 PMC391406

[33] Demeure CE, Brahimi K, Hacini F, Marchand F, Peronet R, et al (2005) Anopheles mosquito bites activate cutaneous mast cells leading to a local inflammatory response and lymph node hyperplasia. J Immunol 174: 3932–3940. 15778349 10.4049/jimmunol.174.7.3932

[34] Kebaier C, Voza T, Vanderberg J (2009) Kinetics of mosquito-injected Plasmodium sporozoites in mice: fewer sporozoites are injected into sporozoite-immunized mice. PLoS Pathog 5: e1000399. 19390607 10.1371/journal.ppat.1000399 PMC2667259

[35] Landau I, Chabaud A (2002) Parasitic pattern of rodent Plasmodium in blood from mouse tail and from Anopheles blood meal. Parassitologia 44: 111–115. 12404818

[36] Diagne M, Petit G, Liot P, Cabaret J, Bain O (1990) The filaria Litomosoides galizai in mites; microfilarial distribution in the host and regulation of the transmission. Ann Parasitol Hum Comp 65: 193–199. 2085265 10.1051/parasite/1990654193

[37] Wilson AB, Clements AN (1965) The Nature of the Skin Reaction to Mosquito Bites in Laboratory Animals. Int Arch Allergy Appl Immunol 26: 294–314. 14291545 10.1159/000229580

[38] Griffiths RB, Gordon RM (1952) An apparatus which enables the process of feeding by mosquitoes to be observed in the tissues of a live rodent; together with an account of the ejection of saliva and its significance in Malaria. Ann Trop Med Parasitol 46: 311–319. 13008362 10.1080/00034983.1952.11685536

[39] Bos JD, Kapsengerg ML (1986) The skin immune system Its cellular constituents and their interactions. Immunology Today 7: 235–240. 25290406 10.1016/0167-5699(86)90111-8

[40] Moon TC, St Laurent CD, Morris KE, Marcet C, Yoshimura T, et al (2010) Advances in mast cell biology: new understanding of heterogeneity and function. Mucosal Immunol 3: 111–128. 20043008 10.1038/mi.2009.136

[41] Owhashi M, Harada M, Suguri S, Omae H, Ishii A (2008) Identification of an eosinophil chemotactic factor from anopheline mosquitoes as a chitinase family protein. Parasitol Res 102: 357–363. 17940798 10.1007/s00436-007-0769-3

[42] Owhashi M, Harada M, Suguri S, Ohmae H, Ishii A (2001) The role of saliva of Anopheles stephensi in inflammatory response: identification of a high molecular weight neutrophil chemotactic factor. Parasitol Res 87: 376–382. 11403380 10.1007/s004360000355

[43] Calvo E, Andersen J, Francischetti IM, de LCM, deBianchi AG, et al (2004) The transcriptome of adult female Anopheles darlingi salivary glands. Insect Mol Biol 13: 73–88. 14728669 10.1111/j.1365-2583.2004.00463.x

[44] Calvo E, Mans BJ, Andersen JF, Ribeiro JM (2006) Function and evolution of a mosquito salivary protein family. J Biol Chem 281: 1935–1942. 16301315 10.1074/jbc.M510359200

[45] Beghdadi W, Porcherie A, Schneider BS, Dubayle D, Peronet R, et al (2008) Inhibition of histamine-mediated signaling confers significant protection against severe malaria in mouse models of disease. J Exp Med 205: 395–408. 18227221 10.1084/jem.20071548 PMC2271011

[46] Amino R, Thiberge S, Martin B, Celli S, Shorte S, et al (2006) Quantitative imaging of Plasmodium transmission from mosquito to mammal. Nat Med 12: 220–224. 16429144 10.1038/nm1350

[47] Sidjanski S, Vanderberg JP (1997) Delayed migration of Plasmodium sporozoites from the mosquito bite site to the blood. Am J Trop Med Hyg 57: 426–429. 9347958 10.4269/ajtmh.1997.57.426

[48] Yamauchi LM, Coppi A, Snounou G, Sinnis P (2007) Plasmodium sporozoites trickle out of the injection site. Cell Microbiol 9: 1215–1222. 17223931 10.1111/j.1462-5822.2006.00861.x PMC1865575

[49] Gueirard P, Tavares J, Thiberge S, Bernex F, Ishino T, et al Development of the malaria parasite in the skin of the mammalian host. Proc Natl Acad Sci U S A 107: 18640–18645. 10.1073/pnas.1009346107 PMC2972976 20921402

[50] Isawa H, Orito Y, Iwanaga S, Jingushi N, Morita A, et al (2007) Identification and characterization of a new kallikrein-kinin system inhibitor from the salivary glands of the malaria vector mosquito Anopheles stephensi. Insect Biochem Mol Biol 37: 466–477. 17456441 10.1016/j.ibmb.2007.02.002

[51] Moreira LA, Saig E, Turley AP, Ribeiro JM, O'Neill SL, et al (2009) Human probing behavior of Aedes aegypti when infected with a life-shortening strain of Wolbachia. PLoS Negl Trop Dis 3: e568. 20016848 10.1371/journal.pntd.0000568 PMC2788697

[52] Turley AP, Moreira LA, O'Neill SL, McGraw EA (2009) Wolbachia infection reduces blood-feeding success in the dengue fever mosquito, Aedes aegypti. PLoS Negl Trop Dis 3: e516. 19753103 10.1371/journal.pntd.0000516 PMC2734393

[53] Mellor AL, Munn DH (2006) Immune privilege: A recurrent theme in immunoregulation? Immunol Rev 213: 5–11.

[54] Taylor MJ, Hoerauf A, Bockarie M (2010) Lymphatic filariasis and onchocerciasis. Lancet 376: 1175–1185. 20739055 10.1016/S0140-6736(10)60586-7

[55] Metenou S, Dembele B, Konate S, Dolo H, Coulibaly SY, et al (2009) Patent filarial infection modulates malaria-specific type 1 cytokine responses in an IL-10-dependent manner in a filaria/malaria-coinfected population. J Immunol 183: 916–924. 19561105 10.4049/jimmunol.0900257 PMC2789677

