# Supplementary figures and images for: Visualizing Non Infectious and Infectious Anopheles gambiae Blood Feedings in Naive and Saliva-Immunized Mice
Source: PLoS One. 2012 Dec 13;7(12):e50464. doi: 10.1371/journal.pone.0050464 (PMC3521732; doi:10.1371/journal.pone.0050464)

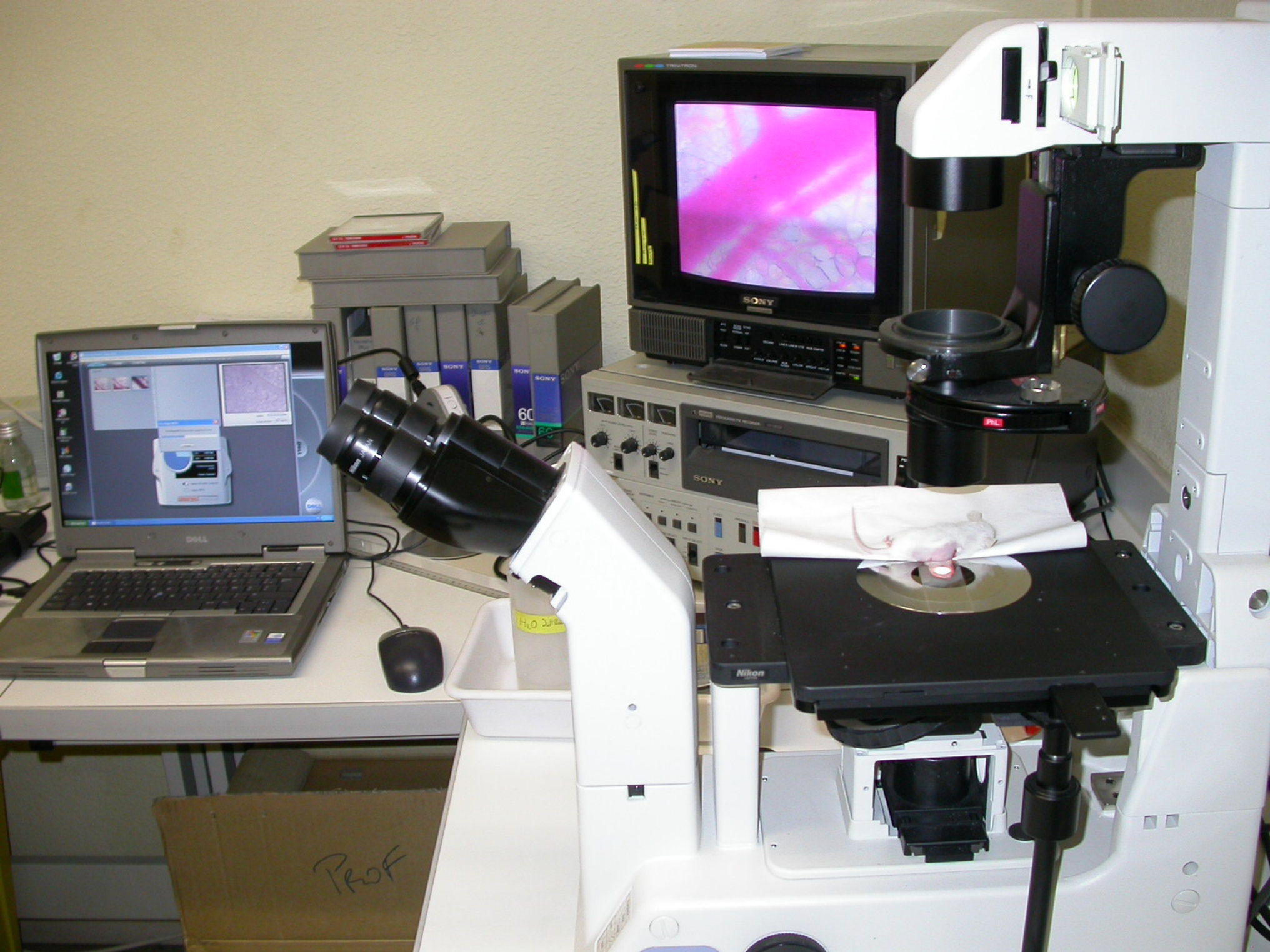

Supplement: Figure S1 — Equipment used for real time blood feeding examination. A Nikon Eclipse TE200 reverse microscope was connected to a digital color video camera. (TIF) [file pone.0050464.s001.tif]

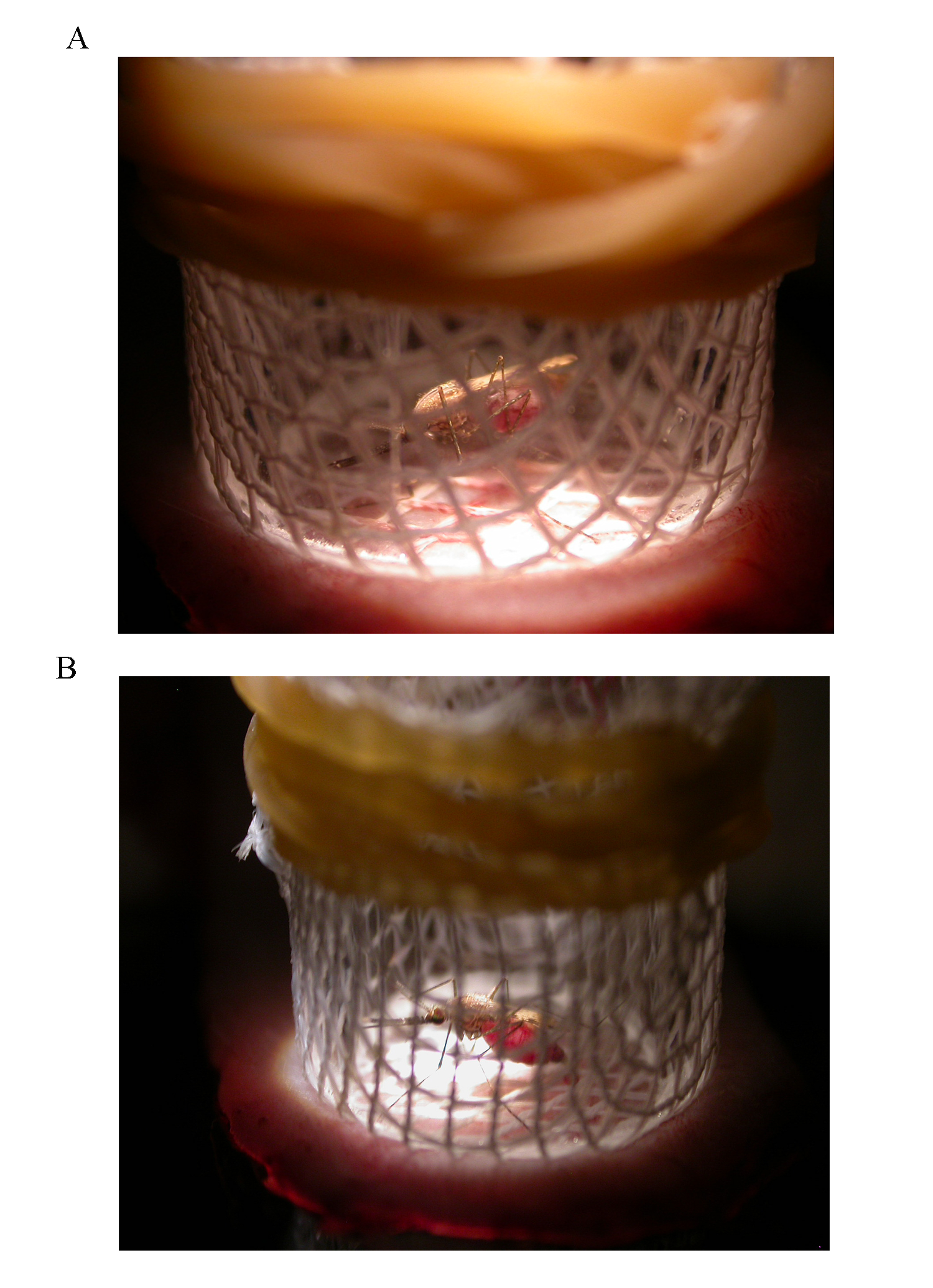

Supplement: Figure S2 — Anopheles gambiae during blood feeding (A) and Anopheles gambiae after blood feeding (B). (TIF) [file pone.0050464.s002.tif]
