# Supplementary material for: Visualizing Non Infectious and Infectious Anopheles gambiae Blood Feedings in Naive and Saliva-Immunized Mice
Source: PLoS One. 2012 Dec 13;7(12):e50464. doi: 10.1371/journal.pone.0050464 (PMC3521732; doi:10.1371/journal.pone.0050464)
Supplement: Table S1 — Comparison between infected and non-infected 23 day-old female mosquitoes (DOC) [file pone.0050464.s016.doc]

Supplementary Table

Supplementary Table 1: Comparison between infected and non-infected 23 day-old female mosquitoes

| **N (%)** | **Non infected**  **(n=9)** | **Infected**  **(n=26)** | **Total**  **(n=35)** | **p** |
| --- | --- | --- | --- | --- |
| Probing | 6 (67) | 11 (42) | 17 (49) | 0.26 |
| Start of probing (s)* | 32 (18-43) | 18 (12-69) | 30 (15-50) | 0.58 |
| Sart of blood feeding (s)* | 409 (250-510) | 240 (236-351) | 260 (236-459) | 0.32 |
| Duration of probing (sec)* | 366 (220-399) | 221 (167-252) | 225 (167-399) | 0.46 |
| Capillary feeding (%) | 4 (44) | 9 (82) | 13 (65) | 0.16 |
| Duration of blood meal (s)* | 340 (270-368) | 405 (300-540) | 340 (300-480) | 0.44 |
| Size of blood vessel* | 2 (1-2) | 1 (1-2) | 1 (1-2) | 0.32 |

**Median (Q1-Q3)*
